# Differences in Functional Connectivity of the Insula Between Brain Wave Vibration in Meditators and Non-meditators

**DOI:** 10.1007/s12671-018-0928-x

**Published:** 2018-03-19

**Authors:** Joon Hwan Jang, Jae-Hun Kim, Je-Yeon Yun, Soo-Hee Choi, Seung Chan An, Do-Hyung Kang

**Affiliations:** 1 Department of Medicine, Seoul National University College of Medicine, Seoul, Republic of Korea; 2 Department of Psychiatry, Seoul National University Hospital, Seoul, Republic of Korea; 3 Department of Radiology, Samsung Medical Center, Sungkyunkwan University School of Medicine, Seoul, Republic of Korea; 4 Yeongeon Student Support Center, Seoul National University College of Medicine, Seoul, Republic of Korea; 5 Department of Psychiatry, Seoul National University College of Medicine, 101 Daehak-ro, Jongno-gu, Seoul, 03080 Republic of Korea; 6 Korea Institute of Brain Science, Seoul, Republic of Korea

**Keywords:** Meditation, Functional magnetic resonance imaging, Insula, Resting-state functional connectivity

## Abstract

The majority of meditation involves focusing attention on internal events or sensations and becoming aware of emotions. The insula cortex, through a functional connection with the prefrontal cortex and other brain regions, plays a key role in integrating external sensory information with internal bodily state signals and emotional awareness. The purpose of this exploratory study was to examine the resting-state functional connectivity of the insula with other brain regions in meditation practitioners and control subjects. Thirty-five Brain Wave Vibration meditation practitioners and 33 controls without meditation experience were included in this study. All subjects underwent 4.68-min resting-state functional scanning runs using magnetic resonance imaging. The anterior and posterior insulae were chosen as seed regions for the functional connectivity map. Meditation practitioners showed significantly greater insula-related functional connectivity in the thalamus, caudate, middle frontal gyrus, and superior temporal gyrus than did controls. Control subjects demonstrated greater functional connectivity with the posterior insula in the parahippocampal gyrus. Our findings suggest that the practice of Brain Wave Vibration meditation may be associated with functional differences in regions related to focused attention, executive control, and emotional awareness and regulation.

**Electronic supplementary material:**

The online version of this article (10.1007/s12671-018-0928-x) contains supplementary material, which is available to authorized users.

## Introduction

Meditation can be conceptualized as a family of complex emotional and attentional regulatory training practices (Lutz et al. 2008). Most types of meditation include focusing attention on internal events or feelings and inhibiting interference from irrelevant external events. Structural and functional neuroimaging studies of meditation have reported some insightful findings in meditation practitioners, suggesting meditation-associated changes in neural circuitry, such as the prefrontal cortex (PFC), anterior cingulate cortex (ACC), striatum, amygdala, and insula (Kang et al. 2013; Luders et al. 2011; Lazar et al. 2005; Jang et al. 2011; Tang et al. 2015).

Previous studies using structural magnetic resonance imaging (MRI) revealed greater cortical density or thickness in the medial PFC or orbitofrontal cortex in meditators compared with non-meditators (Luders et al. 2009; Lazar et al. 2005). Holzel et al. (2008) observed that the gray matter concentration in the medial PFC was correlated with total hours of meditation training in Vipassana meditators. The orbitofrontal cortex and medial PFC are believed to play a role in emotional regulation. A study, examining the long-term effects of meditation on brain structure, found that the thickness of the PFC and insular cortex in Vipassana meditation practitioners was significantly greater than that in controls, which suggests that meditation may influence neural plasticity (Lazar et al. 2005). Greater functional connectivity than controls was also reported within the default mode network in the medial PFC (Jang et al. 2011) and dorsomedial PFC (Taylor et al. 2013). These prefrontal regions receive connections from areas associated with exteroceptive (i.e., perceiving the body’s own position, motion, and state) and interoceptive (i.e., perceiving sensations arising within the body) stimuli. The PFCs also have rich functional connections with the insular cortex, which plays a key role in bodily and emotional awareness, as well as integrating external sensory information with internal bodily state signals and emotion (Craig 2002, 2004; Critchley et al. 2004; Mutschler et al. 2009; Carmichael and Price 1996; Gu et al. 2013).

Brain Wave Vibration (BWV) is a mind-body training designed to focus on bodily sensations, facilitate relaxation, and release negative emotions in the body through natural rhythmic movements. BWV involves focusing attention on one’s bodily sensations and emotion, as well as heightening awareness of the movement of energy within the body. It aims to relax the body and induce positive mind, while the vibrations are believed to tone up the brain arousal. The first step of BWV is to move the body consciously. The second step involves following one’s own natural rhythm and focusing on physical sensations and vibrations, which may spread to all parts of the body. Once the vibration becomes natural and familiar, practitioners reflexively engage in the third step, which is characterized by increased awareness of the movement of energy within the body and a release of negative emotions. BWV has some similarity to yoga practice, as both the practices include distinct forms of postures and breathing exercise (Bowden et al. 2012). Previously, BWV training was shown to significantly reduce stress reduction and improve positive affect (Jung et al. 2010; Lee et al. 2015). BWV practitioners also showed significantly increased default mode network connectivity in the PFC (Jang et al. 2011). Since BWV emphasizes movement and bodily sensation, it is important to consider the connectivity of the insula. However, no previous studies have explored the functional connectivity between the insula and other brain regions in BWV.

A review emphasized the role of the insular cortex in the experience of emotion derived from information about bodily states (e.g., feeling down because of pain) (Uddin et al. 2013). Emotional regulation refers to strategies that can influence emotional awareness, which is controlling which emotions arise and how these emotions are experienced and expressed (Tang et al. 2015). Affective and emotional components from other brain areas are relayed to the insula, and its role involves coordination of these components with other large-scale brain networks (Uddin et al. 2011). Increased interinsular white matter integrity was also reported in Yoga practitioners (Villemure et al. 2014). However, the insular cortex is not functionally homogenous. The posterior insula serves as a primary interoceptive cortex to receive and process direct interoceptive inputs (e.g., the actual intensity of a stimulus) (Frot et al. 2007). The anterior insula is preferentially involved in conscious awareness of interoceptive signals (e.g., the perceived intensity of a stimulus) and the integration of information regarding emotional states (Uddin 2015; Craig et al. 2000). The anterior insula, in conjunction with the dorsolateral PFC, has been implicated in the salience network. Activity in these regions increases in response to various salient stimuli, such as sensory (e.g., pain and temperature) or visceral stimuli (Seeley et al. 2007). These structures are collectively referred to as the fronto-insular cortex (Menon and Uddin 2010; Seeley et al. 2007; Sridharan et al. 2008). However, few studies of the insula-related changes in brain function associated with meditation have been conducted to date.

One strategy for understanding the functional role of a brain region is to focus on its functional connectivity. Resting-state functional connectivity is a functional MRI approach used to evaluate functional interactions among brain regions, which occur when a subject is not performing an explicit task, by analyzing the temporal correlations of spontaneous low-frequency blood oxygen level-dependent (BOLD) signal fluctuations (Fox and Raichle 2007; Fransson 2005). Using this approach, the relationship between anatomically distinct, but functionally connected, brain regions can be efficiently explored (Fox et al. 2005; Fox and Raichle 2007; Bentley et al. 2016). Therefore, considering that most of meditation practices, including BWV, involve the focus of attention on somatic sensations and emotional awareness, investigating the functional connectivity between insular subregions (i.e., the anterior/posterior insula) and other brain areas in experienced meditators could provide new insight into the relation of meditation practice on neuronal network function.

The purpose of the current exploratory study was to investigate the functional role of the insula from the perspective of its functional communications with other brain regions in BWV meditation practitioners to determine the association between meditation and brain function involving emotional and bodily awareness and monitoring. In this study, we performed functional connectivity-based parcellation of the insula using resting-state functional data to determine whether BOLD fluctuations within the anterior and posterior insula correlate with other regions of the brain. We hypothesized that BWV practitioners would show heightened functional connectivity between the insula and the PFC and brain regions associated with sensory processing and emotional awareness and regulation.

## Method

### Participants

Thirty-five meditation practitioners (16 men and 19 women) and 33 control subjects (22 men and 11 women) participated in the current study. The meditation practitioners were recruited from participants of BWV, a type of moving meditation developed in South Korea in the 1980s. BWV is designed to help quiet the thinking mind and release negative emotions by performing specific rhythmic physical movements and focusing on bodily sensations (Bowden et al. 2012; Jung et al. 2010).

The meditation practitioners reported practicing BWV on a daily basis (30–60 min per day) for more than 1 year (mean 39.9 months; range 13–101 months). The control subjects reported no previous experience with meditation or similar practices (e.g., yoga, Tai Chi, or Templestay). The non-patient version of the Structured Clinical Interview for DSM-IV was used to assess psychiatric disorders in all participants. All subjects were right-handed. The exclusion criteria included a known history of psychosis, bipolar disorder, major depressive disorder, substance abuse or dependence, significant head injury, or seizure disorder. The Beck Depression Inventory (BDI) (range 0–17 for meditation practitioners; 0–20 for control subjects) (Beck et al. 1961) and the Beck Anxiety Inventory (BAI) (range 0–14 for meditation practitioners; 0–15 for control subjects) (Beck and Steer 1990) were administered to measure the severity of depression and anxiety, respectively. Age and BDI and BAI scores were normally distributed. There were no differences in age, sex, or BDI or BAI scores between the meditation practitioners and control subjects (Table 1). The present study was approved by the Institutional Review Board of Seoul National University Hospital, and written informed consent was obtained from all subjects.

**Table 1:** Demographic and clinical characteristics of the meditation practitioners and control subjects

	Meditation practitioners	Control subjects	Analysis	
	(*N* = 35)	(*N* = 33)	*T* or *χ*^2^ score	*P* value
Age (year)	25.0 ± 3.5	23.7 ± 3.6	− 1.529	0.131
Sex (M/F)	16/19	22/11	0.082	0.094
Duration of meditation practice (month)	39.9			
BDI score	2.7 ± 6.3	3.0 ± 4.8	0.231	0.818
BAI score	4.4 ± 7.9	3.6 ± 4.2	− 0.545	0.588

Data are given as mean ± standard deviation

*BDI* Beck Depression Inventory, *BAI* Beck Anxiety Inventory

### Procedure

#### Data Acquisition and Preprocessing

Before functional MRI (fMRI) scanning, participants were directed to maintain fixation on a foveal crosshair. Subsequently, participants were explicitly instructed to relax, move as little as possible, and refrain from meditating or thinking of something specific during the fMRI scan. Resting-state functional MRI (rs-fMRI) data processing was carried out using the FMRIB Software Library (FSL) (www.fmrib.ox.ac.uk) and Analysis of Functional Neuroimages (AFNI) (afni.nimh.nih.gov/afni). After discarding the first four images, preprocessing was performed including slice-timing correction, 3D rigid-body translation for head motion correction, and temporal normalization to yield a whole-brain mode value of 1000. Processed rs-fMRI data were temporally band-pass filtered (0.009–0.080 Hz) and spatially smoothed (8-mm full width at half height). Several sources of spurious variance along with their temporal derivatives were then removed from the data through linear regression: six parameters obtained by rigid body correction of head motion, the signal from a ventricular region of interest (ROI), the signal from a region centered in the white matter, and the signal from the brain mask. No participants had head motion of more than 2.0 mm translation in any of the three directions or more than 2.0 maximum rotations around any of the axes during the experiment. In addition, we did not observe any significant difference in motion parameters (average of absolute values across time points) between the meditation and control groups (unpaired two-sample *t* test, *P* < 0.05 for uncorrected multiple comparison).

#### Parcellation of the Insula and Functional Connectivity Maps

**Fig. 1:**
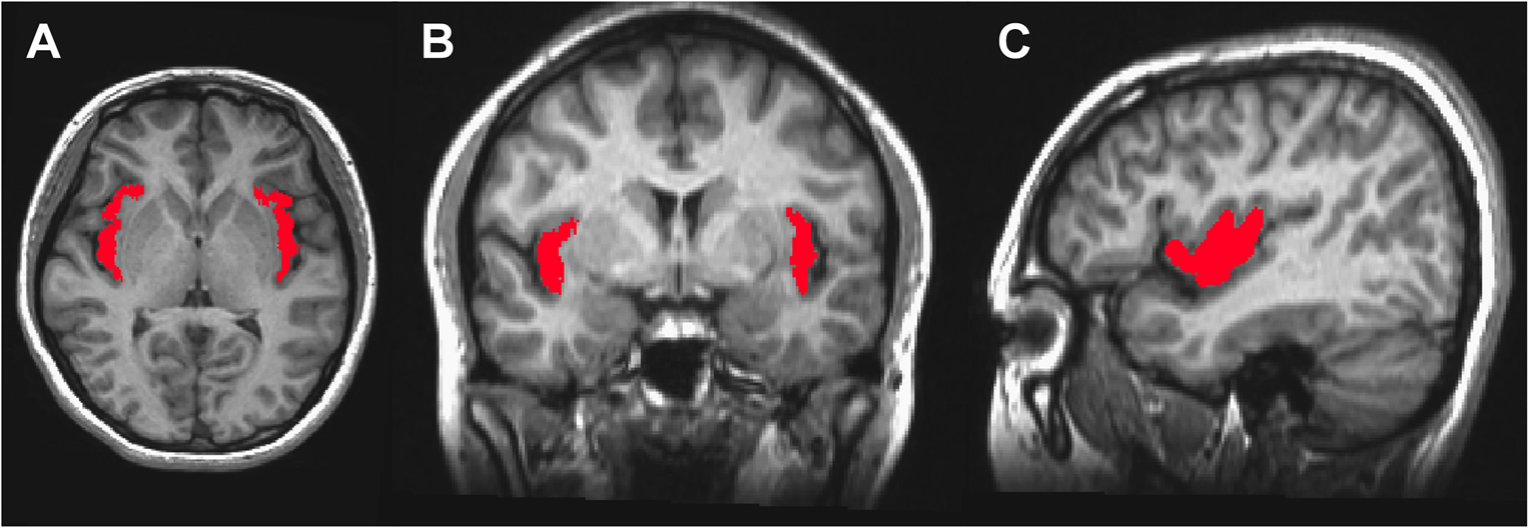
Left and right insular cortex manually defined on T1-weighted MRI in **a** axial, **b** coronal, and **c** sagittal view

In this study, we segmented the insular area into two subregions and computed their functional connectivity maps for each hemisphere using the functional connectivity-based parcellation method (Kim et al. 2010). Briefly, the insular cortex was manually defined on the high-resolution T1-weighted MR image of each hemisphere of each subject (Fig. 1). The manually defined left/right insula mask was spatially normalized into rs-fMRI data. For each rs-fMRI voxel *x* in the left/right insular area, the functional connectivity map *r*_*x*_(*V*), where *V* is the whole-brain set of gray matter voxels, was computed using Pearson’s correlation. The normalized correlation map *Z*_*x*_(*V*) was then computed using Fisher’s *z* transform. The maps were stored in the rows of *Z* (functional connectivity profile matrix) with dimensions of *N*_*x*_ × *N*_*v*_, where *N*_*x*_ is the number of voxels in the left/right insular area and *N*_*v*_ is the number of voxels in the gray matter area. To characterize the degree of similarity between the functional connectivity maps of voxels in the left/right insular area, a functional similarity (*S*) matrix was computed by cross-correlating the *Z* matrix. Each element (*i*, *j*) in the *S* matrix characterizes the degree of similarity between the normalized correlation map *Z*_*i*_(*v*) of the left/right insula voxel and the normalized correlation map *Z*_*j*_(*v*) of the left/right insula voxel. The *K*-means cluster algorithm (*K* = 2) was then applied to segment the left/right insula area into two functional subregions based on the similarity of their functional connectivity (Supplementary Fig. 1). The functional connectivity map for each subregion was computed by averaging across the subset of rows of *Z* corresponding to that cluster. For creating probability maps for the left/right insular subregions and group analysis of functional connectivity related to each subregion of the left/right insula, functionally parcellated insular subregions and their corresponding functional connectivity map for each individual space were spatially transformed into the Montreal Neurological Institute (MNI) space. Within- and between-group analyses were conducted in MNI space (2 × 2 × 2 mm).

### Measures

A 1.5-T Avanto scanner (Siemens, Erlangen, Germany) was used to obtain BOLD signals using an echo-planar imaging sequence for 4.68 min (120 volumes) with the following parameters: TR/TE = 2340/52 ms, FOV = 220 × 220 mm, flip angle = 90°, voxel size = 3.44 × 3.44 × 5.00 mm, and no interslice gap. T1-weighted images were obtained using a three-dimensional magnetization-prepared rapid acquisition gradient-echo (MPRAGE) sequence (TR/TE = 1160/4.76 ms, FOV = 170 × 230 mm, flip angle = 15°, and voxel size = 0.45 × 0.45 × 0.90 mm).

### Data Analyses

An unpaired two-sample *t* test and the chi-square test were used to compare differences in demographic and clinical measures between the two groups. Within-group imaging analyses were conducted using a one-sample *t* test and a false-discovery-rate (FDR) correction threshold of *q* < 0.001. To detect significant differences in the degree of functional connectivity between groups, a permutation test was performed using in-house software written using MATLAB v. 7.6 (Mathworks, Natick, MA). In this test, all participants were randomly assigned to one of two groups, and the distribution of group differences was estimated based on 5000 randomized iterations for each voxel. The voxels, reaching 0.1% (*P* < 0.001, two-tailed) of the estimated distribution of group differences, were deemed statistically significant. To remove the spike-like noisy patterns, not correction for clustering, clusters less than 64 mm^3^ (approximately 1 voxel in the original space) were excluded.

The relationships between Pearson’s correlation coefficients were calculated to investigate the relationships between the duration of meditation practice and BDI/BAI scores and the resting-state functional connectivity strength with the insular cortex. All statistical analyses were two-tailed, with a significance level of probability set at 0.05 (uncorrected for multiple comparison).

## Results

**Fig. 2:**
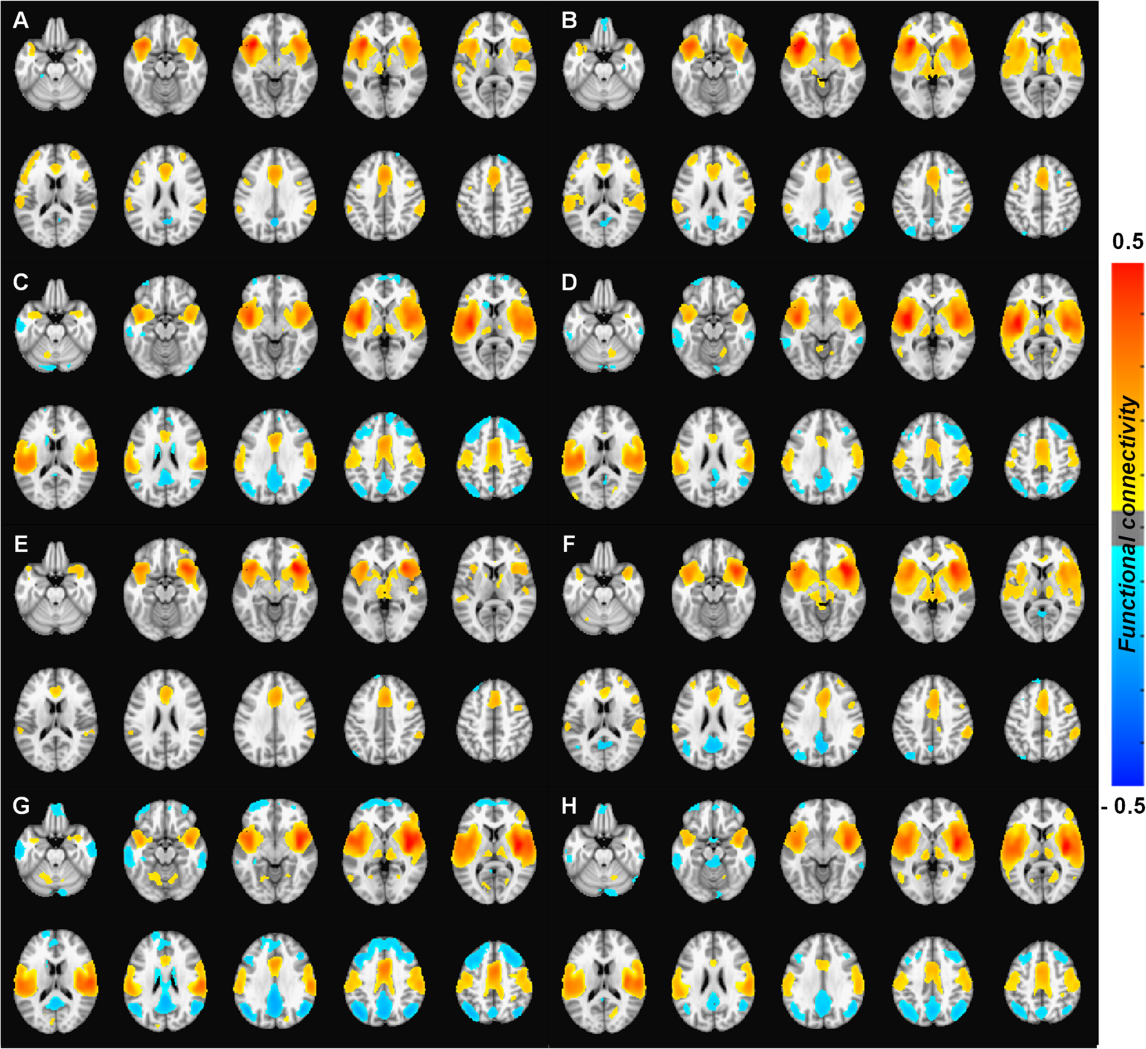
The statistical functional connectivity maps in control group (left panel) and meditator group (right panel) at *P* < 0.001 for multiple comparison (FDR). The statistical functional connectivity maps of the left anterior insular subregion in control group (**a**) and meditator group (**b**) and of the right anterior insular subregions in control group (**e**) and meditator group (**f**). The statistical functional connectivity maps of the left posterior insular subregion in control group (**c**) and meditator group (**d**) and of the right posterior insular subregions in control group (**g**) and meditator group (**h**). The color bar represents the degree of functional connectivity: blue—negative functional connectivity and red—positive functional connectivity

Our results showed that the insular cortex is segmented into anterior and posterior subregions when using the functional connectivity-based parcellation method (Supplementary Fig. 2). The whole-brain functional connectivity maps for the anterior insula in the control group (Fig. 2a/e) and in the meditation practitioners (Fig. 2b/f) showed significant positive functional connections with the bilateral inferior/middle frontal gyrus, inferior parietal lobule, superior temporal gyrus, ACC, pre-supplementary motor areas, thalamus, and caudate. Significant negative functional connections with the bilateral superior frontal gyrus, parahippocampal gyrus, and posterior cingulate were also observed. The whole-brain functional connectivity maps for the posterior insula in the control group (Fig. 2c/g) and in the meditation practitioners (Fig. 2d/h) showed significant positive functional connections with the bilateral pre-/post-central gyrus, superior temporal gyrus, ACC, post-supplementary motor areas, and thalamus, but significant negative functional connections with the bilateral superior/middle frontal gyrus, middle/inferior temporal gyrus, inferior parietal lobule, angular gyrus, posterior cingulate, and caudate.

**Fig. 3:**
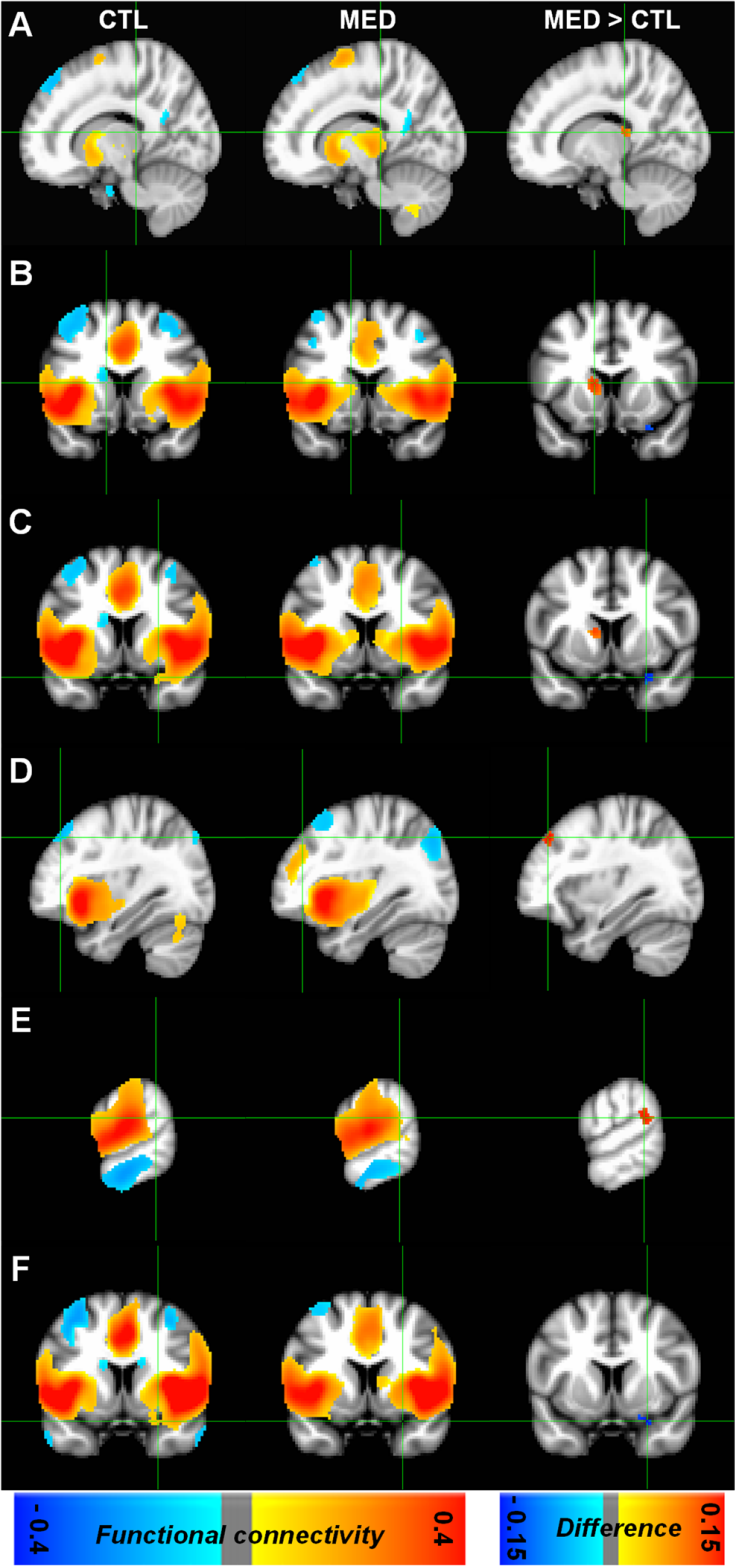
The statistical difference maps between meditation practitioners and control subjects at *P* < 0.001 for multiple comparisons using permutation test. **a** Right thalamus, **b** left caudate, **c** right parahippocampal gyrus, **d** left middle frontal gyrus, **e** left superior temporal gyrus, and **f** right parahippocampal gyrus. The statistical functional connectivity maps in control group (left panel), in meditation group (middle panel), and between-group difference (right panel). In within group analysis (left and middle panel), the red voxels represent the positive functional connectivity and the blue voxels represent the negative functional connectivity at *P* < 0.001 (FDR). In between-group analysis (right panel), red voxels represent the greater functional connectivity and blue voxels represent the lesser functional connectivity in meditation practitioners compared with that in control subjects at *P* < 0.001 (permutation test)

Statistical comparison between the whole-brain functional connectivity group maps showed significant increasing and decreasing functional connectivity between the meditation practitioners and control subjects (Fig. 3). The meditation practitioners showed greater functional connectivity between the dorsolateral PFC (middle frontal gyrus) and thalamus and the anterior insula. In addition, the meditation practitioners also demonstrated greater functional connectivity with the posterior insular cortex in the caudate and the superior temporal gyrus. The meditation practitioners also demonstrated lesser functional connectivity between the posterior insular cortex and the parahippocampal gyrus (Table 2). No significant correlations were found between the functional connectivity with the anterior/posterior insula in the middle frontal gyrus, caudate, thalamus, superior temporal cortex, and parahippocampal gyrus and duration of meditation practice or BDI/BAI scores.

**Table 2:** Brain regions showing difference of functional connectivity with the anterior/posterior insular cortex between meditation practitioners and control subjects (permutation test, *P* < 0.001)

Brain region, Brodmann area	MNI coordinates			Volume (mm^3^)	Max intensity	*P* value
	*x*	*y*	*z*			
MED > CTL (left anterior insula)						
R thalamus	14	− 30	12	352	0.108	4.0 × 10^−7^
MED > CTL (left posterior insula)						
L caudate	− 12	12	10	1088	0.124	4.5 × 10^−5^
MED < CTL (left posterior insula)						
R parahippocampal gyrus, 28	26	8	− 24	352	− 0.138	4.0 × 10^−7^
MED > CTL (right anterior insula)						
L middle frontal gyrus, 9/8	− 32	38	42	416	0.122	6.0 × 10^−7^
MED > CTL (right posterior insula)						
L superior temporal gyrus, 40/22	− 62	− 46	20	464	0.140	2.0 × 10^−5^
MED < CTL (right posterior insula)						
R parahippocampal gyrus, 28	26	8	− 22	304	−0.146	1.0 × 10^−6^

*FDR* false-discovery-rate, *R* right, *L* left

## Discussion

In the present study, we found that meditation practitioners appear to have significantly greater functional connectivity with the anterior insula in the thalamus and PFC, as well as with the posterior insula in the caudate and superior temporal gyrus. We also observed lesser functional connectivity in the meditation practitioners between the posterior insula and the parahippocampal gyrus. A posterior-to-anterior gradient in the insular cortex has been proposed, in which features of interoception are processed in the posterior insula, whereas integration with emotional and cognitive information occurs in the anterior insula. Thus, the insula is believed to form an interoceptive image of one’s physical state and, as a result, to play a crucial role in subjective awareness of emotions (Craig 2002; Gu et al. 2013).

Meditation has been thought to be complex emotional regulation training techniques developed for emotional balance and cultivation of well-being (Lutz et al. 2008). Bowden et al. (2014) reported improvements in mood and sense of well-being after BWV practice. Our finding of increased functional connectivity between the anterior insula and thalamus and the PFC are in line with previous studies (Manuello et al. 2016; Tang et al. 2015; Laneri et al. 2016). A recent study using diffusion tensor imaging found that meditation practitioners demonstrated higher fractional anisotropy values in the white matter connected to the insula and thalamus, which may be interpreted as enhanced white matter integrity or increased structural connectivity (Laneri et al. 2016). One of the functions of the thalamus is to relay sensory information to the cerebral cortex including the insula. Gu et al. (2013) also provided evidence showing that the anterior insula appears to play a role in emotional awareness. The anterior insula integrates bottom-up interoceptive sensory signals with top-down predictions to generate a current emotional awareness state (Gu et al. 2013). Therefore, the greater functional connection between the anterior insula and thalamus could be related to more efficient focused emotional attention through meditation practice. This may occur through the changed patterns of temporal interaction in thalamic cells after meditation (Saggar et al. 2015).

The PFC is functionally and anatomically connected to the insula (Sridharan et al. 2008; Craig 2009). The fronto-insular cortex has been shown to initiate switching signals that activate the central executive network and deactivate the default mode network during cognitively demanding tasks (Sridharan et al. 2008). Meditation practice has been associated with increased activation in areas involved in sustaining and monitoring the focus of attention, including the dorsolateral PFC, and with increased between-network functional connectivity between the dorsolateral PFC and the insula (Mooneyham et al. 2016). Increased functional connectivity of the PFC may contribute to enhanced intermodular communication between the executive control network and the salience network (Mooneyham et al. 2016; Tomasino and Fabbro 2016). Allen et al. (2012) demonstrated that mindfulness intervention improved executive control accompanied by increased BOLD changes in the dorsolateral PFC.

It has been proposed that the caudate is associated with transmission of anxiety and drive, as a component of the reward system, as well as executive function such as a goal-directed action (Guehl et al. 2008; Grahn et al. 2008). There is also evidence to suggest that the caudate is involved in attentional processes, in particular response inhibition (Aron and Poldrack 2005). A structural MRI study demonstrated that the gray matter density of the caudate nucleus was increased after meditation training in patients with Parkinson’s disease (Pickut et al. 2013). Monti et al. (2012) reported increased cerebral blood flow in the caudate and insula among other regions after an 8-week mindfulness-based program. Additionally, the degree of increased cerebral blood flow in the left caudate was significantly correlated with decreased self-reported levels of anxiety. In another recent study, neural activation in the caudate and anterior insula was attenuated in meditation practitioners during reward anticipation (Kirk et al. 2015), proposing that meditation practitioners are less susceptible to monetary incentives and related subjective psychological distress (Kirk et al. 2015; Kirk et al. 2011). Moreover, Buddhist meditation practitioners showed elevated activity in the somatosensory cortex and superior temporal cortex, which suggests that meditation practitioners activate brain networks enabling them to uncouple a negative emotional response to an unfair offer (Kirk et al. 2011). Meditation practice might enable the subject to better modulate anxiety and decision-making through awareness of negative thoughts and emotions and in turn accepting them, but not attaching or reacting to them (also called “decentering”), which may enhance a subjective sense of well-being (Jung et al. 2016; Creswell et al. 2007). However, further studies are warranted to understand the implications of decreased negative arousal on decision-making and well-being.

In this study, we observed lesser functional connectivity between the posterior insula and the parahippocampal gyrus in meditation practitioners. The parahippocampal gyrus, in conjunction with the medial PFC and insula, comprises the paralimbic system. The paralimbic system is important for communication between the limbic system and neocortex and is associated with emotional regulation, self-projection, and different aspects of memory encoding and retrieval in declarative long-term memory (Schacter et al. 1999; Viard et al. 2011). Activation of the parahippocampal gyrus was observed during episodic memory retrieval and rest (Stark and Squire 2001). The parahippocampal gyrus also has a strong connection with the amygdala, which is associated with anxiety and negative emotions (Stein et al. 2007). In another study, this connection was increased significantly during emotional versus neutral film viewing (Kilpatrick and Cahill 2003). Therefore, weaker functional connectivity between the parahippocampal gyrus and the posterior insula may be related to the release of negative emotion, one of the training procedures of BWV. In a structural MRI study, altered gray matter volume in the parahippocampal gyrus was observed in loving-kindness meditators (Leung et al. 2013).

## Limitations

There are several limitations to the present study. First, because of the cross-sectional nature of this study, we could not explore the longitudinal causal direction of influence. This introduces the important confound that people who decide to practice meditation may differ from others at baseline with respect to psychological and cultural backgrounds, or even brain activation and structural traits. The cross-sectional design, with the relatively small sample size in each group, may also explain the absence of significant correlations between functional connectivity and duration of BWV practice. Second, whether this study, which included meditation practitioners who have practiced BWV, can be generalized to other kinds of meditation practice, especially mindfulness meditation or focused attention meditation, is questionable. Different kinds of meditation would be expected to have different patterns of functional connectivity that vary according to their approach. Future studies are needed to compare differences among the diverse techniques of meditation. Another limitation is the problem of reverse inference (Poldrack 2011). Thus, it could be interpreted cautiously to attribute the difference in functional connectivity of certain brain regions to specific cognitive function.

## Electronic Supplementary Material

Supplementary Fig. 1 Functional connectivity-based parcellation of the left insular mask. (A) The manually defined left insula mask was spatially transformed into EPI space. (B) For left insular mask, functional connectivity profile (Z) matrix was computed for every voxel in left insular mask with the gray level voxels in the brain. (C) The functional similarity (S) matrix was obtained by cross-correlation of the Z matrix. (D) The reordered functional similarity matrix was created using K-means clustering algorithm. (E) The left insular mask was segmented into anterior and posterior subregions based on the functional connectivity patterns. (GIF 2359 kb)

High Resolution Image (TIFF 4363 kb)

Supplementary Fig. 2 The probability maps of the functionally segmented anterior insular subregions for the left hemisphere in the control subjects (A) and the meditation practitioners (B), and for the right hemisphere in the control subject (E) and the meditation practitioners (F). The probability maps of the functionally segmented posterior insular subregions for the left hemisphere in the control subjects (C) and the meditation practitioners (D), and for the right hemisphere in the control subject (G) and the meditation practitioners (H). The color bar represents the overlapping sub-regions for 33 control subjects and 35 meditation practitioners. (GIF 972 kb)

High Resolution Image (TIFF 2390 kb)
