# Supplementary figures and images for: Differences in Functional Connectivity of the Insula Between Brain Wave Vibration in Meditators and Non-meditators
Source: Mindfulness (N Y). 2018 Mar 19;9(6):1857–66. doi: 10.1007/s12671-018-0928-x (PMC6244630; doi:10.1007/s12671-018-0928-x)

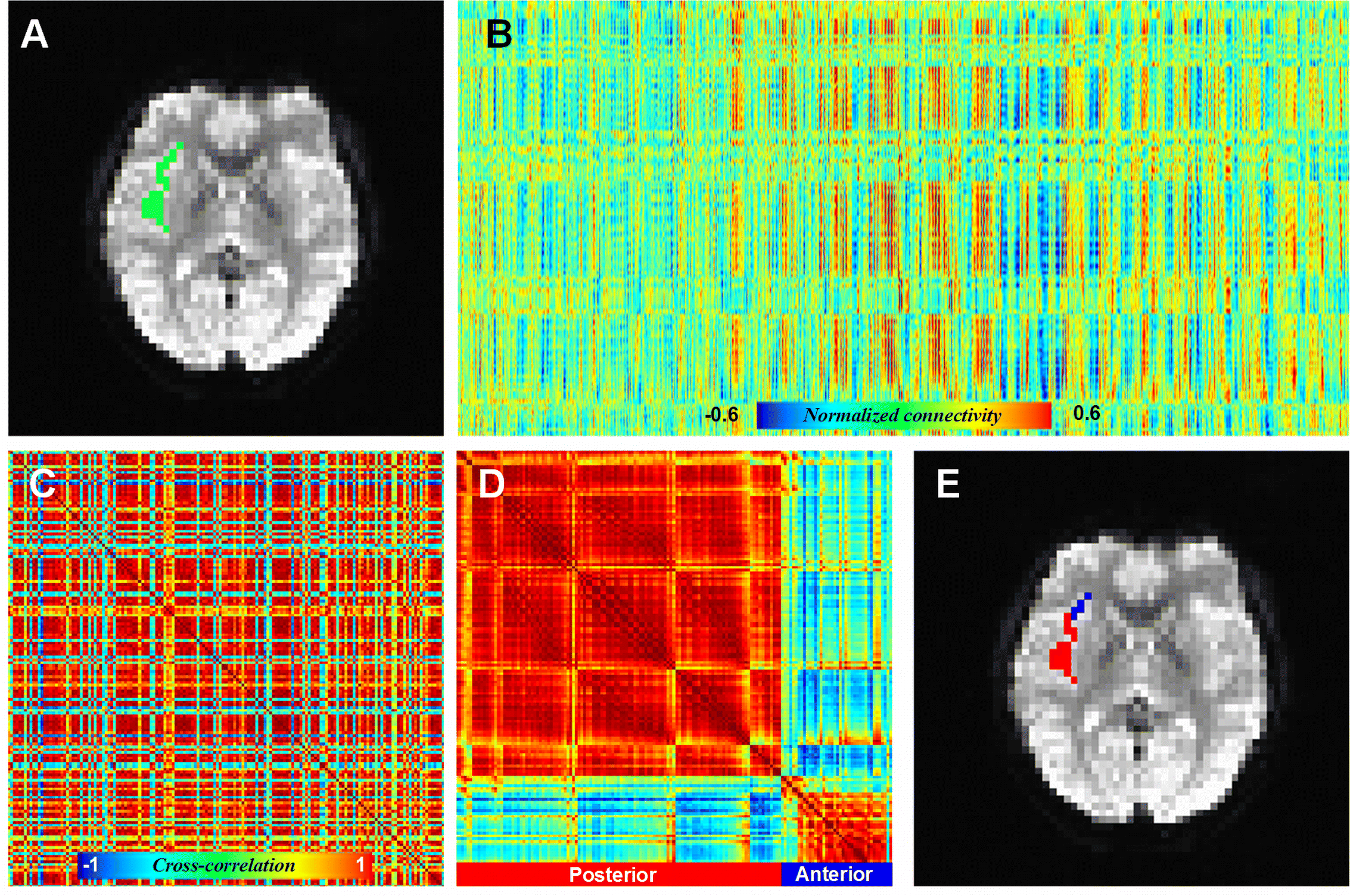

Supplement: Supplementary file 1 — Functional connectivity-based parcellation of the left insular mask. (A) The manually defined left insula mask was spatially transformed into EPI space. (B) For left insular mask, functional connectivity profile (Z) matrix was computed for every voxel in left insular mask with the gray level voxels in the brain. (C) The functional similarity (S) matrix was obtained by cross-correlation of the Z matrix. (D) The reordered functional similarity matrix was created using K-means clustering algorithm. (E) The left insular mask was segmented into anterior and posterior subregions based on the functional connectivity patterns. (GIF 2359 kb) [file 12671_2018_928_Fig4_ESM.gif]

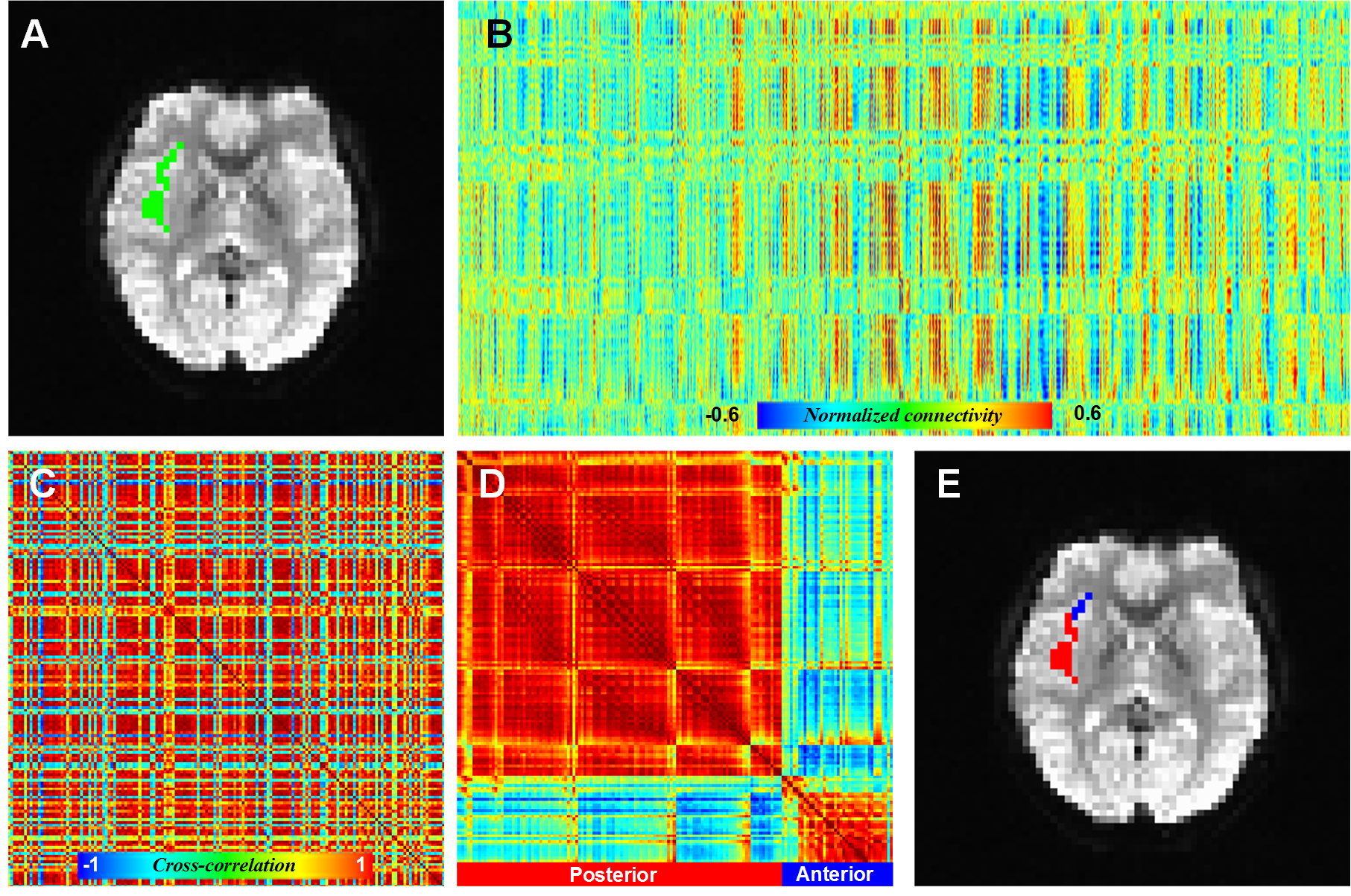

Supplement: Supplementary file 2 — High Resolution Image (TIFF 4363 kb) [file 12671_2018_928_MOESM1_ESM.tif]

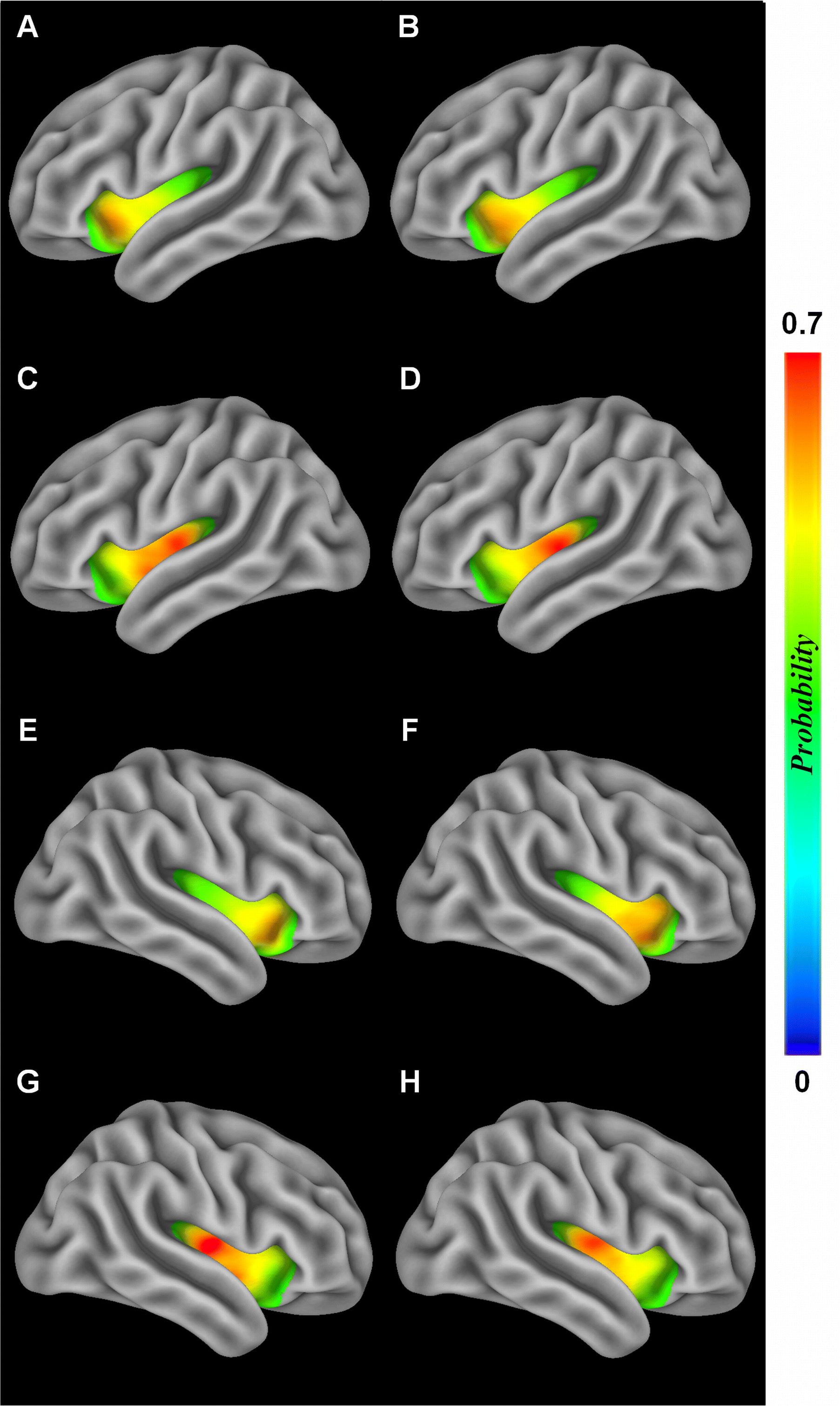

Supplement: Supplementary file 3 — The probability maps of the functionally segmented anterior insular subregions for the left hemisphere in the control subjects (A) and the meditation practitioners (B), and for the right hemisphere in the control subject (E) and the meditation practitioners (F). The probability maps of the functionally segmented posterior insular subregions for the left hemisphere in the control subjects (C) and the meditation practitioners (D), and for the right hemisphere in the control subject (G) and the meditation practitioners (H). The color bar represents the overlapping sub-regions for 33 control subjects and 35 meditation practitioners. (GIF 972 kb) [file 12671_2018_928_Fig5_ESM.gif]

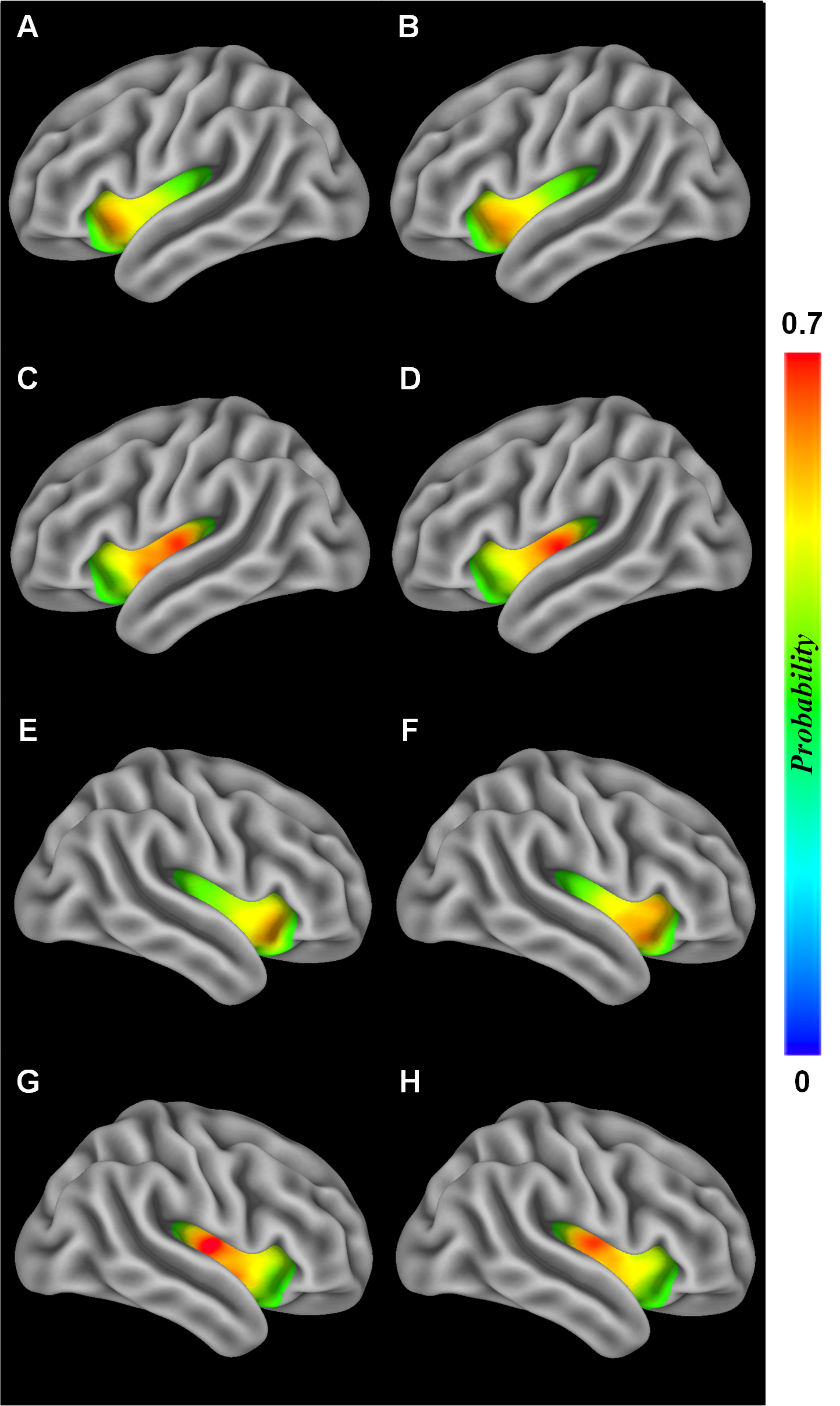

Supplement: Supplementary file 4 — High Resolution Image (TIFF 2390 kb) [file 12671_2018_928_MOESM2_ESM.tif]
